# Characteristics of the antibiotic regimen that affect antimicrobial resistance in urinary pathogens

**DOI:** 10.1186/s13756-018-0368-3

**Published:** 2018-06-18

**Authors:** Boudewijn Catry, Katrien Latour, Robin Bruyndonckx, Camellia Diba, Candida Geerdens, Samuel Coenen

**Affiliations:** 1 Healthcare-associated infections & Antimicrobial resistance (https://www.nsih.be), Sciensano, Ruy Juliette Wytsmanstraat 14, Brussels, 1050 Belgium; 2 Department of Public Health and Primary Care, KU Leuven - University of Leuven, Leuven, Belgium; 3 Interuniversity Institute for Biostatistics and statistical Bioinformatics (I-BIOSTAT), Hasselt University, Hasselt, Belgium; 4 Laboratory of Medical Microbiology, Vaccine & Infectious Disease Institute (VAXINFECTIO), University of Antwerp, Antwerp, Belgium

**Keywords:** Route of administration, Drug resistance, Uropathogens, Elderly

## Abstract

**Background:**

Treatment duration, treatment interval, formulation and type of antimicrobial (antibiotic) are modifiable factors that will influence antimicrobial selection pressure. Currently, the impact of the route of administration on the occurrence of resistance in humans is unclear.

**Methods:**

In this retrospective multi-center cohort study, we assessed the impact of different variables on antimicrobial resistance (AMR) in pathogens isolated from the urinary tract in older adults. A generalized estimating equations (GEE) model was constructed using 7397 *Escherichia coli* (*E. coli*) isolates.

**Results:**

Resistance in *E. coli* was higher when more antibiotics had been prescribed before isolation of the sample, especially in women (significant interaction *p* = 0.0016) and up to nine preceding prescriptions it was lower for higher proportions of preceding parenteral prescriptions (significant interactions *p* = 0.0067). The laboratory identity, dying, and the time between prescription and sampling were important confounders (*p* < 0.001).

**Conclusions:**

Our model describing shows a dose-response relation between antibiotic use and AMR in *E. coli* isolated from urine samples of older adults, and, for the first time, that higher proportions of preceding parenteral prescriptions are significantly associated with lower probabilities of AMR, provided that the number of preceding prescriptions is not extremely high (≥10 during the 1.5 year observation period; 93% of 5650 included patients).

**Trial registration:**

Retrospectively registered.

**Electronic supplementary material:**

The online version of this article (10.1186/s13756-018-0368-3) contains supplementary material, which is available to authorized users.

## Background

The bacterium *Escherichia coli (E. coli)* is by far the most common uropathogen in older adults [1]. Investigations in residents from long-term care facilities also revealed that the primary indication for antimicrobial (antibiotic) use is a urinary tract infection (UTI) [2]. If a lower UTI spreads to the kidneys or, via a blood stream infection, to other organs, life threatening organ failure can occur [3].

An antimicrobial therapy consists of a specific product, synergies with other agents, its route of administration (formulation), a dose, a treatment interval, treatment duration, and they all can have an effect on the selection of antimicrobial resistance (AMR) [4, 5]. A vast amount of studies has been focusing on synergies, the ideal dose (pharmacokinetic/pharmacodynamic parameters [6]), treatment interval and the impact of duration on resistance [7], to maintain clinical efficacy while minimizing resistance. In contrast, limited research has been done on the importance of the route of administration on the occurrence of resistance. The purpose of the present research was to study the influence of different variables of the antimicrobial prescription on the occurrence of resistance in *E. coli* isolated from urine samples in Belgian older adults (≥65 years).

## Methods

### Data

Microbiological results for individual patient samples, retrieved from 15 voluntary participating clinical laboratories (2005) were linked with individual antimicrobial consumption and sociodemographic data (July 2004 – December 2005). The latter were retrieved from the Intermutualistic Agency (IMA), which bundles national reimbursement information from the seven Belgian health insurance funds. These data were collected within a large retrospective cohort study assessing the link between antimicrobial consumption and resistance in the individual patient [8]. In the current study, we focused on the resistance status (i.e. susceptible versus non-susceptible) of *E. coli* isolates found in the urine of retired adults (aged 65 or above) in relation to the consumption of antibacterials for systemic use (substances with Anatomic Therapeutic Chemical (ATC) code J01) [9]. Patients for whom antimicrobial consumption data were available but no urine sample was analyzed, or for whom a sample was analyzed but no antimicrobials were prescribed during the study period, were excluded for the here described analysis.

Antimicrobial susceptibility testing results for *E. coli* were obtained from Kirby Bauer disk diffusion tests with a wide variety of number and agents examined. The majority of labs applied Clinical Laboratory Standards Institute (CLSI) guidelines for inoculum standardization, incubation conditions and breakpoint interpretation criteria. An isolate’s resistance status (Antimicrobial Resistance Iindex; ARI) was calculated as the number of non-susceptible test results divided by the total number of antimicrobials tested (expressed as the proportion of non-susceptible test results) [10]. Antimicrobial consumption was summarized as the total dose of prescribed antimicrobials (expressed as the number of defined daily doses; DDD), the number of unique preceding prescriptions (N_prescriptons) and the proportion of unique preceding prescriptions for a parenteral antimicrobial (%Injectable). Antimicrobial agents had to be purchased minimally 2 days before the sample was taken to ensure that patients started taking the purchased antibiotic at the moment of sampling. Prescriptions for the same antimicrobial (identical ATC level 4 code) within 7 days were considered as one unique prescription. Other covariates that were considered are gender (male or female), age category (65–84 or 85 and above), whether the patient died during the year of the study or was still alive at the end of 2005 (yes or no; death), and the log(time). For the log(time), the logarithmic value of the time was calculated, with time defined as the number of days between sampling and the last prescription. Previous antimicrobial consumption was not restricted to antimicrobials only prescribed for urinary tract infections.

### Statistical analysis

Because multiple samples from the same patient were potentially taken, observations within the same patient are expected to be correlated. To account for the correlated nature of the data, a generalized estimating equations (GEE) model [11] was used. Because the explanatory covariates are time-dependent, we used an independent working correlation [12]. Note that although this working correlation might be incorrect, parameter estimates and empirical standard errors are deemed consistent due to the use of a sandwich estimator [13]. A GEE model with ARI as the outcome variable and a logit link function was constructed. To account for the fact that one lab analyzed multiple samples and determined the number of antibiotics tested, we included laboratory identification code (Lab ID) as a covariate in the GEE model. Because the remaining covariates considered to explain antimicrobial resistance were numerous (7 covariates and their two-way interactions), we conducted model building in two steps. In a first step, we removed all insignificant (*p* > 0.15) covariates in a backward fashion. In a second step, we included significant two-way interactions between remaining covariates in a forward fashion, using α = 0.05. Due to collinearity between the dose and the number of preceding prescriptions (Pearson correlation = 0.73), we decided to continue with the latter.

### Ethics statement

Data from laboratories and reimbursement organizations were encrypted by a trusted third party to ensure patient confidentiality. The procedure and the study protocol were approved by the Sectorial committee of the Belgian Federal Social Security as well as by the jointed ethical committee of the Scientific Institute of Public Health (WIV-ISP) and the Centres for Veterinary and Agrochemical Research (CODA-CERVA) (both institutes merged on April 2018 into Sciensano).

## Results

The final data used in this study contained information on resistance status for 7397 isolates retrieved from 5650 patients (Table 1). The majority of patients were female (79%), were aged 65–84 years (76.8%) and survived 2005 (82.2%). The number of isolates per patient widely varied (Additional file 1: Table S1) and the ARI showed differences according to the gender, partly related to the different compounds tested (Additional file 2: Figure S1).

**Table 1:** Characteristics of the antimicrobial prescriptions in 5650 older adults prior to (minimum 2 days) an isolation of *Escherichia coli* (*n* = 7379) from a urine sample as retrieved from 15 voluntary participating Belgian clinical laboratories (January 2005 – December 2005)

Variable	Men (1551 isolates)		Women (5846 isolates)	
	Median	IQR	Median	IQR
Time	24	[10–79]	41	[13–125]
DDD	27.8	[10.3–64.1]	23	[9.5–53.0]
N_prescriptions	4	[2–6]	3	[2–6]
%Injectable	25	[0–57]	11	[0–50]
ARI *E. coli*	0.17	[0–0.35]	0.13	[0–0.31]

*IQR*: interquartile range *Time*: time in days between sampling and start of preceding antimicrobial (antibiotic) prescription. *DDD*: sum of defined daily dose (DDD) prior to sampling. *N_prescriptions*: number of prescriptions (If the same antimicrobial formulation (substance) was delivered within 7 days this was defined as one prescription). *%Injectable*: route of administration (modeled as the ratio of preceding injectable over preceding orally administered antimicrobial prescriptions, i.e. the proportion of preceding parenteral prescriptions). *ARI*: Antimicrobial Resistance Index calculated as proportion of non-susceptible antimicrobial resistance test results as defined by Kirby Bauer disk diffusion test

### Descriptive statistics of prescribed antibiotics

Table 1 shows the antimicrobial prescriptions reported in the cohort. The mean (standard deviation) number of prescriptions and DDD per patient was 4.4 (4.1), and 44.4 (59.2), respectively (see for extra information Additional file 1: Table S2).

### Statistical model building

Backward model building (using α = 0.15) resulted in the inclusion of covariates related to gender, log(time), the number of preceding prescriptions, route of administration (modeled as the proportion of non-oral antimicrobials prescribed), the lab in which the isolates were analyzed and whether or not the patient survived 2005. Subsequent forward model building (using α = 0.05) resulted in the inclusion of the interactions between the number of preceding prescriptions on the one hand and the proportion of non-oral antimicrobials prescribed or the patient’s gender on the other hand. The odds ratios (95% Wald confidence intervals) of the final model are reported in Table 2.

**Table 2:** Odds ratios (95% Wald confidence intervals) for covariates in the final model* that determine antimicrobial resistance (higher Antimicrobial Resistance Index, ARI) in *Escherichia coli* from retired patients that have been prescribed antimicrobials at least 2 days prior to sampling

Co-variate	Odds ratio [95%CI]	Co-variate	Odds ratio [95%CI]
Gender (male)	1.29 [1.14–1.45]	Log(time)	0.83 [0.81–0.85]
N_prescriptions	1.05 [1.04–1.06]	Survival (yes)	0.84 [0.78–0.92]
%Injectable	1.00 [0.99–1.00]	N_prescriptions * %Injectable	1.0004 [1.0001–1.0007]
		N_prescriptions * gender (male)	0.97 [0.956–0.99]

N_prescriptions: number of preceding antimicrobial prescriptions received 2 days or more before each sample; %Injectable: proportion of parenteral (non-oral) preceding antimicrobial prescriptions. *Laboratory identity (*n* = 15) was controlled for in the final model (*p* < 0.0001), but individual values were not included in the Table

**Fig. 1:**
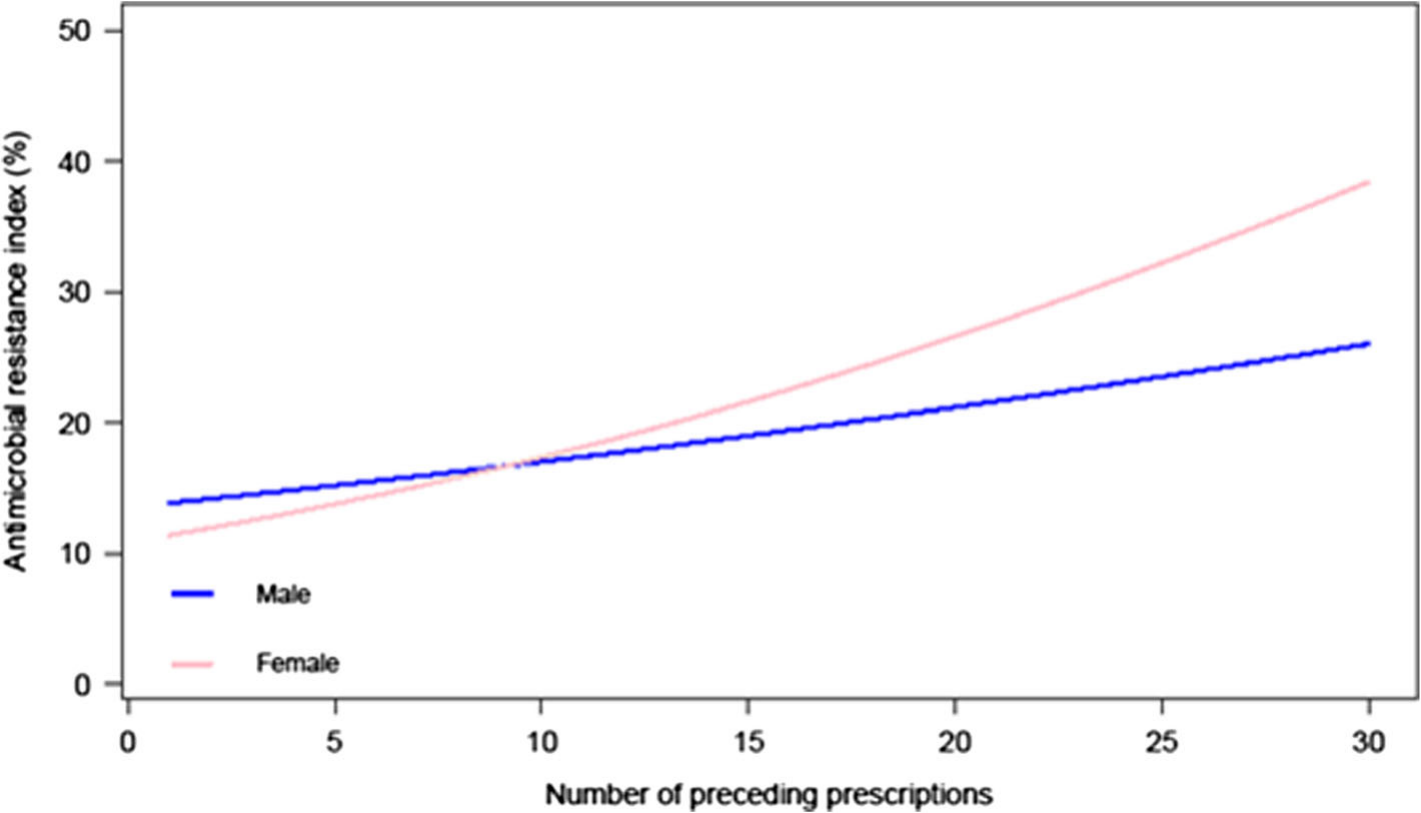
Predicted antimicrobial resistance index (ARI) as reported for *Escherichia coli* isolated from the urinary tract of retired patients (Belgium, 2005) when varying the number of preceding prescriptions (1–30) for male and female patients. Estimates were obtained from a generalized estimating equations (GEE) model (fitted for a patient that was alive at the end of the study and was tested 33 (median) days after the most recent prescription in reference laboratory 15)

**Fig. 2:**
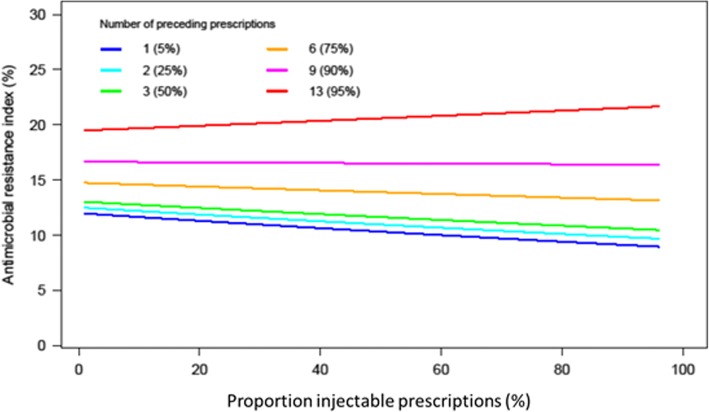
Probability of resistance as estimated by the antimicrobial resistance index (ARI) as reported for *Escherichia coli* isolates from urinary tract infections in retired patients (Belgium 2005), when varying the proportion of injectable (% non-oral) prescriptions and the number of preceding prescriptions. Estimates were obtained using the final generalized estimating equations (GEE) model (fitted for a female patient that was alive at the end of the study and was tested 33 (median) days after her antibiotic prescription in lab 15)

The final model revealed that the odds of resistance (non-susceptibility, i.e. a higher ARI) decreased when the patient was still alive at the end of 2005 and when time between sampling and prescribing increased. The formulation (%Injectable; proportion of preceding parenteral prescriptions) effect depended on the number of preceding prescriptions and the patient’s gender. When the number of preceding prescriptions is below 10, the odds of non-susceptibility is higher for men. When the number of preceding prescriptions is high (> 9), the odds of non-susceptibility is higher for women (Fig. 1). As seen in Fig. 2, if the number of preceding prescriptions is below 10, the predicted ARI is lower for a higher proportion of preceding parenteral prescriptions. If the number of preceding prescriptions is high (> 9), the predicted ARI is lower for a lower proportion of preceding parenteral prescriptions. In other words, up to nine preceding prescriptions the higher the proportion of preceding parenteral prescription the lower the odds for AMR. When exposed to more than nine preceding prescriptions this effect is no longer present (calculated over the time frame of 1.5 year).

## Discussion

This retrospective multicenter study showed a dose-response relationship between antimicrobial use and resistance in uropathogens in older adults. Our results demonstrate, for the first time in human clinical isolates, that the oral route of administration is associated with an increased likelihood of resistance compared to the parenteral route, provided the number of prescriptions (week courses) is below 10 (over one and a half year observation time). This is in full agreement with animal experimental studies in rodents for *E. coli* exposed to betalactams and tetracyclines [14], and earlier findings in a randomized control field trial in cattle [15]. The effect of route of administration moreover interacted with the number of preceding prescriptions. Up to 9 prescriptions, when other variables held constant, probability of resistance decreased by increase in proportion of preceding parenteral antibiotic prescriptions. Since, seemingly, resistance gets organized after some threshold, possibly by reorganization of resistance at the molecular level, a different pattern was observed for samples with more than 9 prescriptions.

### Comparison with the literature

Recently, a study comparing resistance in faecal *E. coli* from different groups of children (healthy, cancer, cystic fibrosis), suggested that aminopenicillin administered intravenously had only a modest effect on selection of intestinal resistance in cancer patients and possibly less impact than oral administration, which was the main route of administration of aminopenicillin to children with cystic fibrosis [16].

Apart from these studies, relatively little attention has been recently given to the route of administration and its particular influence on antimicrobial resistance. One exception is the stimulation to switch from intravenous to oral formulations (IV/PO switch) as soon as possible in acute care hospitals to reduce length of stay, treatment costs, and central line associated infections [17]. Our results should trigger research to examine the influence of this switch on the selection of antimicrobial resistance. A recent investigation in Switzerland has shown that in acute care practice such a switch, leads to a two-step broad spectrum selection pressure, with an oral exposure of predominantly amoxicillin clavulanic acid, and to a lesser extent fluoroquinolones or clindamycine [4]. This impact on resistance in pathogens and commensals thus is substantial and we therefor plea to examine this in defined case control settings and larger at the population level. Infection control intervention studies likewise should include antimicrobial resistance data of pathogens, preferably over consecutive years [18]. Of notice, the IV/PO switch rationale is also fundamentally contradictory to the general mutation prevention theory. This theory states that a short high (loading) dose, followed by regular dosing intervals during an as short as possible time period is able to minimizes the resistance selection pressure while maintaining clinical efficacy [6, 19, 20]. Our study was conducted to assess such dynamics and persistence for *E. coli* retrieved from urine samples in the older adults (retired population). We assume many of these patients were suspected or confirmed to have a urinary tract infection. For urinary tract infections in women, a Cochrane review published in 2002 has shown that a reduction of treatment duration is feasible without impairing clinical efficacy and therefore should be encouraged to minimize the development and spread of resistance [21]. This is in line with our observations when using the number of week courses as a proxy for treatment duration and should further be stimulated in general and specialized practice.

The urinary tract mostly gets infected with *E. coli* by retrograde infection from commensal faecal bacteria. Each time an inappropriate antimicrobial therapy is initiated in the individual patient resistant genes can be selected. Other risk factors for developing drug resistant UTI include previous antimicrobial exposure, long-term care residence, older age and comorbidities such as diabetes [2]. Dutch investigators have also identified other medication and diet, including animal derived food, to be a risk factor for resistance in bacteria involved in UTI [22]. Bacteria can obtain antimicrobial resistant genes either by mutation or by acquisition f romneighboring bacteria (horizontal transfer). This has formerly been investigated and well documented for *E. coli*, both as a commensal [10, 23] and an invasive pathogen organism (e.g. EARS-net). This resistance selection process and maintenance after withdrawal of antibiotic pressure (i.e. persistence) can further be stimulated or driven by unrelated antimicrobial agents (co-selection) [23]. Despite that minimal inhibitory concentration determinations are the golden standard, under routine laboratory conditions, in *E. coli* and many other fast growing organisms, disk diffusion tests have for long been the method to simultaneously determine susceptibility profiles for a wide variety of antimicrobial agents. For these reasons, the antimicrobial resistance index (ARI, [10, 24]) was used as primary outcome variable. It can theoretically even take subtle changes in the antibiogram into account, and merges selection pressure effects of virtually all antimicrobial agents used including co-selection by unrelated organisms. It has been shown to be strongly correlated with treatment incidences expressed as prescribed or administered daily dosages (PDD & DDD) at different population levels and settings [24–26]. Causal relationships between DDD and antimicrobial resistance have been found in single center longitudinal studies [27].

It was observed that patients who died during the study period were on average, more likely to have strains that were resistant to antibiotics. This is in line with similar observations in other bacteria [8]. The effect of number of days between the sample and the last prescription (log(time)) was significant and negative; indicating that probability of resistance is higher in the days after the treatment and decreases over time, confirming earlier findings in bacteria retrieved from the respiratory tract [28]. We further observed a high variability in ARI across participating laboratories and this demands further research in terms of validation of antimicrobial resistance surveillance.

### Strengths and limitations

The study has several limitations, like the voluntary participation of the laboratories, the reliability of the(ir) disk diffusion tests, the lack of information on co-morbidities of the patients, the applied dosage assessment [25] and the absence of compliance information with regard to the prescribed antibiotics, and the unknown selection criteria related to patients that undergo laboratory examinations of the urinary tract. Selection bias due to inter-laboratory and gender driven differences in the panel of antimicrobial agents tested also could have influenced the analysis. Deviations in dosage regimens that might interfere with the resistance selection could not be identified with the applied methodology. Also patients not receiving antibiotics were excluded in the current study design. The latter information could be used to assess a baseline level of resistance as our study group earlier explored for the respiratory tract system [28]. Since also antimicrobial agents prescribed for indications other that urinary tract infections were included in the analysis it seems reasonable to conclude that resistance selection pressures are not restricted to one organ system, given the potential effect of antimicrobial agents on the digestive tract [14] and thereby indirectly on organisms shed by stool that can cause urinary tract infections. Also all prescriptions were considered in our analysis because of co-selection due to linked resistance genes as demonstrated for *E. coli* [23]. An additional confounder that potentially could have driven the selection between oral or injectable administrations is the difference between empirical, prophylactic and microbiologically directed regimes. In a European study in long term care facilities executed in 2009 [2], empirical treatments were most common (54.4%), followed by prophylactic (28.8%) and microbiologically documented (16.1%) regimes [2]. It is also recommended to repeat the analysis to confirm the finding by prospective randomized and controlled studies and in other study populations. Moreover, undesired effects of the switch of formulations, as recommended by several international guidelines on antimicrobial stewardship, should be considered in further studies.

## Conclusion

In conclusion, this multicenter retrospective cohort study demonstrated a clear dose-effect of antimicrobial prescriptions on resistance in *E. coli* routinely isolated in urine samples from older adults. A substantial effect of route of administration, though subject to the number of preceding prescriptions, on the occurrence of antimicrobial resistance in uropathogens was demonstrated.

## Additional files

Additional file 1: **Table S1.** Distribution of *Escherichia (E.) coli* isolates (*n* = 7379) per patient (retired, *n* = 5650) retrieved from 15 voluntary participating Belgian clinical laboratories (January 2005 – December 2005), for which an antimicrobial was prescribed (minimum 2 days before sampling) during the study period (July 2004–December 2005). **Table S2** Average number of defined daily dose (DDD) by gender prior to the isolation of uropathogens from retired patients (*n* = 5650) in Belgium (2004–2005). (DOCX 16 kb)

Additional file 2: **Figure S1.** Exhaustive distribution of antimicrobial susceptibilities stratified by patients’ gender (left) and age (right) category, as reported for *Escherichia (E.) coli* isolates retrieved from urinary tract infections in Belgium (2005). (TIF 66 kb)

## Abbreviations

AMR: antimicrobial resistance
ARI: Antimicrobial resistance index
ATC: Anatomic therapeutic chemical
CLSI: Clinical & Laboratory Standards Institute
CODA-CERVA: Veterinary & Agrochemical Research Centre (currently Sciensano)
DDD: Defined daily dose
*E. coli*: *Escherichia coli*
EARS-Net: European Antimicrobial Resistance Surveillance-Network
GEE: Generalized estimating equations
IMA: Intermutualistic agency
Injectable%: The proportion of parenterally administered (non-oral) antimicrobials
IV/PO: Intravenously/per os
Lab ID: Laboratory identification code
N_prescriptions: The number of unique prescriptions
PDD: Prescribed daily dose
UTI: Urinary tract infection
WIV-ISP: Scientific Institute of Public Health (currently Sciensano)
