# Supplementary material for: Characteristics of the antibiotic regimen that affect antimicrobial resistance in urinary pathogens
Source: Antimicrob Resist Infect Control. 2018 Jun 18;7:76. doi: 10.1186/s13756-018-0368-3 (PMC6006702; doi:10.1186/s13756-018-0368-3)
Supplement: Supplementary file 1 — Table S1. Distribution of Escherichia (E.) coli isolates (n = 7379) per patient (retired, n = 5650) retrieved from 15 voluntary participating Belgian clinical laboratories (January 2005 – December 2005), for which an antimicrobial was prescribed (minimum 2 days before sampling) during the study period (July 2004–December 2005). Table S2 Average number of defined daily dose (DDD) by gender prior to the isolation of uropathogens from retired patients (n = 5650) in Belgium (2004–2005). (DOCX 16 kb) [file 13756_2018_368_MOESM1_ESM.docx]

Additional file 1

**Table S1.** Distribution of *Escherichia (E.) coli* isolates (n= 7379) per patient (retired, n=5650) retrieved from 15 voluntary participating Belgian clinical laboratories (January 2005 – December 2005), for which an antimicrobial was prescribed (minimum two days before sampling) during the study period (July 2004-December 2005) .

| No. of isolates | No. of patients |  | No. of isolates | No. of patients |
| --- | --- | --- | --- | --- |
| 1 | 6129 |  | **6** | 10 |
| 2 | 1095 |  | **7** | 10 |
| 3 | 254 |  | **8** | 3 |
| 4 | 101 |  | **9** | 3 |
| 5 | 19 |  | **10** | 1 |

**Table S2**: Average number of defined daily dose (DDD) by gender prior to the isolation of uropathogens from retired patients (n=5650) in Belgium (2004-2005).

| ATC group | Male  Mean (SD) | Female  Mean (SD) |
| --- | --- | --- |
| J01C | 11.35 (12.59) | 11.13 (13.51) |
| J01D | 5.29 (8.10) | 6.47 (15.36) |
| J01E | 9.01 (7.83) | 9.78 (9.33) |
| J01M | 7.42 (6.38) | 7.27 (5.55) |
| J01X | 14.23 (14.74) | 14.43 (13.89) |
| Other | 7.71 (6.21) | 7.64 (6.07) |
| Total | 9.11 (10.65) | 9.37 (12.39) |

ATC: Anatomical Therapeutic Chemical [9]: J01C: Beta-lactam antibacterials, Penicillins group; J01D: other Beta-lactam antibacterials; J01E: Sulphonamides and trimethoprim; J01M: quinolones; J01X: other antibacterials including glycopeptides, metronidazole, and polymyxines.
