# Supplementary figures and images for: Characteristics of the antibiotic regimen that affect antimicrobial resistance in urinary pathogens
Source: Antimicrob Resist Infect Control. 2018 Jun 18;7:76. doi: 10.1186/s13756-018-0368-3 (PMC6006702; doi:10.1186/s13756-018-0368-3)

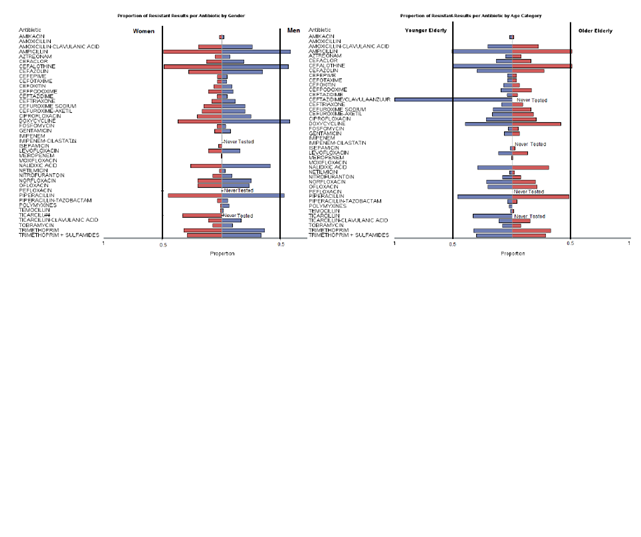

Supplement: Supplementary file 2 — Figure S1. Exhaustive distribution of antimicrobial susceptibilities stratified by patients’ gender (left) and age (right) category, as reported for Escherichia (E.) coli isolates retrieved from urinary tract infections in Belgium (2005). (TIF 66 kb) [file 13756_2018_368_MOESM2_ESM.tif]
